# Regulatory T Cells Inhibit T Cell Activity by Downregulating CD137 Ligand via CD137 Trogocytosis

**DOI:** 10.3390/cells10020353

**Published:** 2021-02-09

**Authors:** Khang Luu, Mugdha Vijay Patwardhan, Qun Zeng, Stina L. Wickström, Andreas Lundqvist, Herbert Schwarz

**Affiliations:** 1 Department of Physiology, Yong Loo Lin School of Medicine, National University of Singapore, Singapore 117593, Singapore; khangluu@u.nus.edu (K.L.); mugdha.p@u.nus.edu (M.V.P.); zengqun@u.nus.edu (Q.Z.); 2 NUS Immunology Programme, Life Sciences Institute, National University of Singapore, Singapore 117456, Singapore; 3 NUSMED Immunology Translational Research Programme, National University of Singapore, Singapore 117456, Singapore; 4 Integrative Sciences and Engineering Programme, National University of Singapore, Singapore 117456, Singapore; 5 Department of Oncology-Pathology, Karolinska Institutet, 171 64 Stockholm, Sweden; stina.wickstrom@ki.se (S.L.W.); Andreas.Lundqvist@ki.se (A.L.)

**Keywords:** regulatory T cells, trogocytosis, CD137

## Abstract

CD137 is a costimulatory molecule expressed on activated T cells. CD137 ligand (CD137L) is expressed by antigen presenting cells (APC), which use the CD137—CD137L system to enhance immune responses. It was, therefore, surprising to discover CD137 expression on regulatory T cells (Treg). The function of CD137 in Treg are controversial. While some studies report that CD137 signalling converts Treg to effector T cells (Teff), other studies find that CD137-expressing Treg display a stronger inhibitory activity than CD137^-^ Treg. Here, we describe that CD137 on Treg binds to CD137L on APC, upon which one of the two molecules is transferred via trogocytosis to the other cell, where CD137—CD137L forms a complex that is internalized and deprives APC of the immune-stimulatory CD137L. Truncated forms of CD137 that lack the cytoplasmic domain of CD137 are also able to downregulate CD137L, demonstrating that CD137 signalling is not required. Comparable data have been obtained with human and murine cells, indicating that this mechanism is evolutionarily conserved. These data describe trogocytosis of CD137 and CD137L as a new mechanism employed by Treg to control immune responses by downregulating the immunostimulatory CD137L on APC.

## 1. Introduction

The costimulatory molecule CD137 is a main driver for cellular immune responses. Cross-linking of CD137 strongly enhances proliferation, IFN-γ secretion, and the cytolytic activity of T cells. CD137 costimulation enables the immune system to eliminate tumours as shown in a wide plethora of murine tumour models [1,2,3,4]. Agonistic anti-CD137 antibodies also strongly enhance antivirus immune responses [5,6,7]. 

Given that CD137 signalling is a potent driver of cellular immune responses, it is surprising to find strong expression of CD137 not only on Teff but also on Treg. In fact, CD137 was found to be a marker for Treg. Human and murine Tregs that had infiltrated tumours, expressed high amounts of CD137 [8] and a high frequency of CD137^+^ Treg correlated with poor prognosis in lung adenocarcinoma patients [9]. This is in line with CD137^+^ Treg being more suppressive than CD137^-^ Treg [10,11]. It is, however, not clear how CD137 expression helps Treg to become more suppressive.

It was postulated that CD137 signalling enhances Treg activity similarly as it enhances Teff activity. Zhang et al., 2007, reported that an agonistic anti-CD137 antibody promoted the proliferation of Treg, which maintained Foxp3 expression and the ability to suppress conventional CD4^+^ T cells [12]. Similarly, Elpek et al., 2007, found that Treg up-regulate CD137 expression in response to IL-2 and that CD137 stimulation expands Treg. These Treg were characterized by increased expression of CD25, CD137, and TGF-β, and they not only suppressed T cell proliferation but also prevented the rejection of allogeneic islets [13]. Recently, Lubrano et al., 2020, reported that CD137 stimulation activates Treg via the NF-κB pathway [14]. However, the hypothesis that CD137 signalling enhances Treg activity is contradicted by other findings, which demonstrate that CD137 agonists inhibit Treg functions and Treg differentiation. In a Friend virus infection model, agonistic CD137-specific antibodies made CD8^+^ T cells resistant to Treg-mediated suppression but did not affect the suppressive function of the Treg [15]. Further, CD137 costimulation inhibited antigen- and TGF-β-driven conversion of naïve CD4^+^ FoxP3^−^ T cells to induced Treg, via the stimulation of IFN-γ production by the naive CD4^+^ T cells [16]. Moreover, CD137 costimulation has been shown to convert a subset of CD4^+^ Foxp3^+^ Treg to Teff that were able to eliminate tumour cells [17].

While most of the studies agree that CD137 is expressed by Treg and that CD137^+^ Treg exert a stronger suppressive activity than CD137^−^ Treg, there are opposing views on the functional role of CD137 signalling into Treg.

Our current study attempts to solve this contradiction by demonstrating that Treg use CD137 to downregulate the immunostimulatory CD137L on APC by trogocytosis and subsequent internalization. This activity is conserved between human and mouse.

The concept of phagocytosis, where one cell engulfs another cell, and pinocytosis, where one cell ingests liquid, are well understood. However, trogocytosis is a comparatively new concept. Trogocytosis refers to one cell nibbling off pieces from another cell [18]. The outcome on the target cells could range from the removal of cell surface molecules, to the induction of cell death. The acceptor cells can digest the acquired material or display it as its own, a process called cross-dressing. For example, basophils were shown to acquire MHC-II molecules from dendritic cells, which endowed them with the ability to present antigens and to stimulate T cells [19].

While the mechanism of trogocytosis is only partly understood, it is clear that trogocytosis can be facilitated by specific ligand-receptor interactions. For instance, Treg use CTLA-4 to remove CD80 and CD86 from APC, thus depriving the APC of the ability to costimulate T cells, a process termed transendocytosis [20].

Here, we show that Treg can use CD137 in a similar process to remove the costimulatory CD137L from APC and thereby to tune down T cell activation.

## 2. Materials and Methods

### 2.1. Generation of Modified CD137 and CD137L Constructs

Murine CD137L was tagged with orange fluorescent protein (OFP) at the N-terminus by incorporating the murine CD137L sequence into pCMV3-N-OFPSpark vector (Sino Biological, Wayne, PA, USA). Murine full-length and truncated CD137 were tagged with green fluorescent protein (GFP) at the C-terminus by incorporating the sequence with or without the cytoplasmic domain into pCMV3-C-GFPSpark vector (Sino Biological).

Full-length human CD137 tagged with GFP was generated in the vector pcDNA3.1(+) by adding GFP to the carboxy-terminus (cytoplasmic domain) of CD137 with a flexible linker (GGGGS)_3_. By designing primers amplifying this entire plasmid except for the cytoplasmic domain of CD137, truncated human CD137, tagged with GFP, was generated using the Q5^®^ Site-Directed Mutagenesis Kit (New England Biolabs, Ipswich, MA, USA).

### 2.2. Cell Culture

The human B cell lines Daudi and Raji and the human monocytic cell line THP-1 were cultured in RPMI-1640 medium (Gibco, Thermo Fisher Scientific, Waltham, MA, USA) supplemented with 10% foetal bovine serum (FBS; Gibco).

The mouse macrophage cell line RAW264.7 (ATCC), the adherent Chinese hamster ovary (CHO), and human HEK293 (HEK; InvivoGen, San Diego, CA, USA) cell lines were cultured in high glucose Dulbecco’s Modified Eagle Medium (DMEM; Biowest Nuaillé, France) supplemented with 10% FBS.

A stable knockout of mouse CD137L in the RAW264.7 cell line was done with CRISPR/Cas9 with the gRNA sequence TCTGAGGAGCGCCGCATCCG cloned into the eSpCas9-2A-GFP plasmid (GenScript, Piscataway, NJ, USA). Cells were electroporated with the plasmid using the Neon transfection system (Thermo Fisher Scientific) and selected by several rounds of FACS for CD137L-negative cells.

To generate CD137L-OFP RAW264.7 cells, a plasmid containing CD137L-OFP was introduced into CD137L-deficient RAW264.7 cells by electroporation, using the SF Cell Line 4D-Nucleofector™ X Kit (Lonza, Basel, Switzerland) and the program DS-136, according to the manufacturer’s instruction. The electroporated cells were cultured for 24 h, and the expression of CD137L-OFP was confirmed by microscopy and flow cytometry.

HEK cells stably expressing fluorescent full-length human CD137 (HEK-FL-CD137-GFP) and truncated human CD137 (HEK-TR-CD137-GFP) were established by lentiviral transduction. To produce the lentiviruses, HEK cells were transfected with equal molarity of the packing plasmids (pRSV-Rev and pMDLg/pRRE from Addgene: Watertown, MA, USA), the envelop plasmid (pMD2.G from Addgene), and lentiviral transfer plasmids. Furthermore, 6 h after transfection, the medium was changed to fresh medium. Supernatants containing virus particles were collected on the first and the second day. Cell debris was removed by pelleting supernatants at 800 g for 10 min. Supernatants containing lentiviruses were aliquoted and stored in −80 °C. For the transduction step, HEK cells were incubated with virus-containing supernatants supplemented with 8 μg/mL polybrene (Sigma-Aldrich, St Louis, MO, USA) overnight and selected with several rounds of FACS to select for fluorescent cells.

CHO cells transiently expressing fluorescent CD137 or CD137L were obtained by transfecting CHO cells with the respective plasmids, using TurboFect™ (Thermo Fisher Scientific) according to the manufacturer’s instruction.

Where indicated, cells were stained with Carboxyfluorescein-succinimidyl-ester (CFSE), (BioLegend, San Diego, CA, USA) or CellTrace™ Violet (Thermo Fisher Scientific) before the coculture, as per manufacturer’s instruction.

Blood products were purchased from the Karolinska Blood Bank from healthy volunteers who provided written and informed consent. Human peripheral blood mononuclear cells (PBMC) were isolated by density gradient centrifugation using Ficoll-Paque PLUS (GE Healthcare, Chicago, IL, USA).

### 2.3. Cell Sorting and Isolation 

Murine T cells were FACS-sorted from wild-type (WT) or CD137^−/−^ C57BL/6 murine splenocytes according to surface markers: CD4^+^CD25^−^ (conventional T cells (Tcon)) or CD4^+^CD25^+^ (Treg). Human Tregs and CD8^+^ T cells were isolated from healthy donor PBMC by CD4^+^CD25^+^CD127^dim/−^ Regulatory T Cell Isolation Kit II and CD8^+^ T Cell Isolation Kit, respectively (Miltenyi Biotec, Bergisch Gladbach, Germany).

### 2.4. T Cell Costimulation Assay

THP-1 cells were cocultured with HEK WT, HEK-FL-CD137 GFP, and HEK-TR-CD137 GFP at a ratio of 1 THP-1 cell to 10 HEK cells, for 1 h. THP-1 cells were then isolated from the coculture by MACS using CD45 Microbeads (Miltenyi Biotec). THP-1 cells were then fixed with BD Cytofix/Cytoperm solution (BD Biosciences, San Jose, CA, USA) at a concentration of 10^6^ cells/mL for 15 min at room temperature, washed with sterile protein buffer saline (PBS) twice, and resuspended in growth medium. Furthermore, 5 × 10^3^ fixed THP-1 cells were then cocultured with 5 × 10^4^ isolated CD8^+^ T cells for 72 h, in the presence of 0.5 µg/mL of anti-CD3 antibody (clone OKT3, BioLegend). The growth media were collected for IFN-γ ELISA (Mabtech, Nacka Strand, Sweden), and the cells were stained for CD25 expression.

### 2.5. Confocal Imaging

Live cell imaging of the coculture was performed with the Olympus FV1000 confocal laser scanning microscope (Tokyo, Japan) at 60× water lens setting.

### 2.6. Flow Cytometry

To determine the expression of cell surface markers, cells were blocked with human Fc receptor (FcR) blocking reagent (Miltenyi Biotec) or Human TruStain FcX™ (BioLegend) and then stained with fluorochrome-conjugated antibodies diluted in flow cytometry staining buffer (PBS containing 2% FBS). Anti-CD137-PE (clone 4B4-1; BD Biosciences), anti-CD137L-APC (clone 5F4; BioLegend) or anti-CD137L-BV421 (clone 5F4; BioLegend), anti-CD45-PE/Dazzle CF594 (clone HI30; BioLegend) or anti-CD45-APC-H7 (clone 2D1; BD Biosciences), anti-CD25-BV421 (clone M-A251; BioLegend), anti-PD-1-APC (clone MIH4; eBioscience, Thermo Fisher Scientific) as where indicated. To determine the expression of mouse cell surface markers, cells were blocked with mouse FcR blocking reagent (Miltenyi Biotec) and stained with the following antibodies diluted in flow cytometry staining buffer: Anti-CD4-BUV395 (clone GK1.5, BD Biosciences) or anti-CD4-eFlour 450 (clone RM4-5, Thermo Fisher Scientific), anti-CD25-PE (clone PC61.5, Thermo Fisher Scientific) or anti-CD25-FITC (clone PC61.5.3, Thermo Fisher Scientific), anti-CD137-APC (clone 17B5; BioLegend), and anti-CD137L-PE (clone TKS-1; BioLegend). To determine cell viability, cells were stained with DAPI (Sigma-Aldrich) or LIVE/DEAD™ Fixable Violet Dead Cell Stain (Thermo Fisher Scientific) or LIVE/DEAD™ Fixable Near-IR Dead Cell Stain (Thermo Fisher Scientific).

### 2.7. Statistical Analysis

Statistical significance was determined by a two-tailed unpaired Student’s *t*-test, or one-way ANOVA with Dunnett multiple comparison test, in GraphPad Prism software. The statistics were calculated from at least three replicates. The graphs show the mean ± standard deviation (SD). A *p* value less than 0.05 indicates statistical significance.

## 3. Results

### 3.1. Murine Tregs Use CD137-Mediated Trogocytosis to Downregulate CD137L on APC

**Figure 1:**
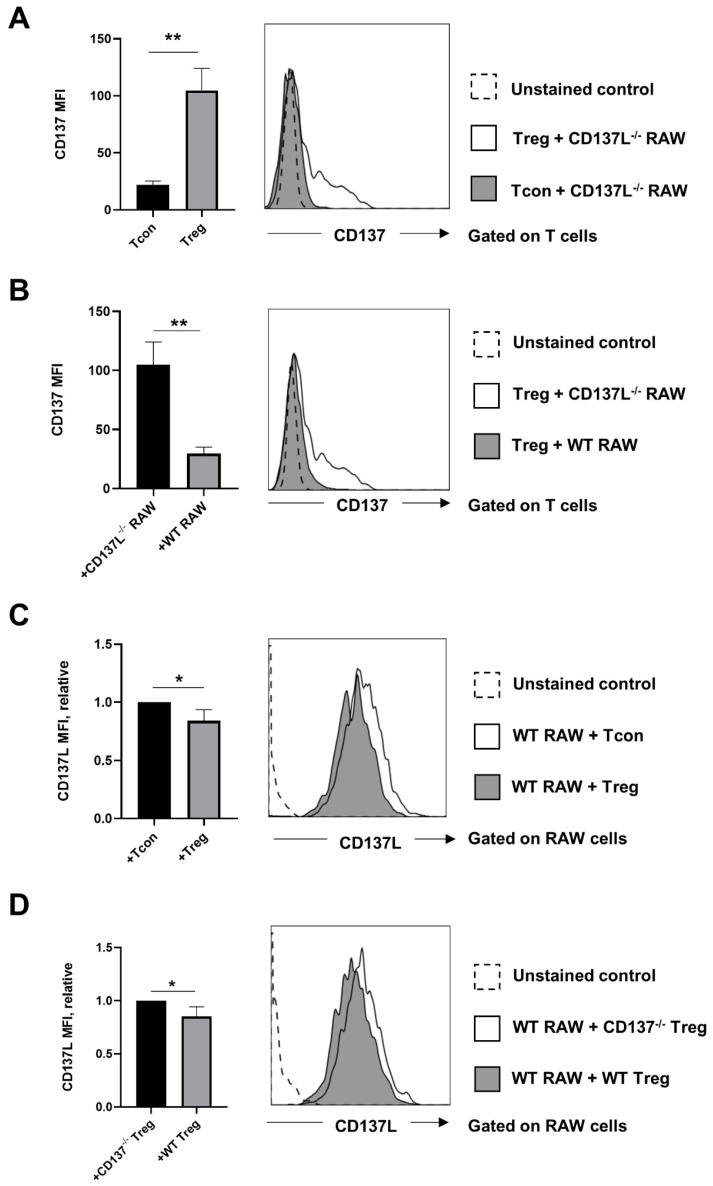
Transfer of CD137 and CD137 ligand (CD137L) between murine regulatory T cells (Treg) and antigen presenting cells (APC). Results were obtained after 1 h of coculture at a 1:1 ratio. (**A**) CD137L-deficient (CD137L^‒/‒^ ) RAW cells were cocultured with either wild-type (WT) conventional T cells (Tcon) or WT Treg. Graph shows mean fluorescence intensity (MFI) of CD137 on Tregs and Tcons, after the coculture. (**B**) WT Treg were cocultured with WT RAW264.7 or CD137L^‒/‒^ RAW cells. Graph shows MFI of CD137 on Tregs, after the coculture. (**C**) WT RAW264.7 cells were cocultured with WT Tcon or WT Treg. Graph shows relative CD137L MFI on WT RAW 264.7 cells after the coculture. (**D**) WT RAW264.7 cells were cocultured with WT Treg or CD137-deficient (CD137^‒/‒^) Treg. Graph shows relative CD137L MFI on WT RAW 264.7 cells after the coculture. All graphs show means ± SD, * *p* < 0.05, ** *p* < 0.01, two-tailed unpaired Student’s *t* test. Data are representative of three independent experiments.

To test whether CD137-CD137L trogocytosis plays a role in immune regulation by Treg, we FACS-sorted Treg and Tcon from murine splenocytes, and cocultured them with the murine macrophage cell line RAW264.7, which expresses high levels of CD137L. As a control, we cocultured the sorted T cells with CD137L-deficient RAW264.7 cells (CD137L^−/−^ RAW) (Supplementary Figure S1A). We found that Treg express more CD137 than Tcon, which is in line with previous reports [11] (Figure 1A). When murine Treg were cocultured with RAW264.7 cells, CD137 levels on Treg decreased, and this decrease was dependent on the presence of CD137L as shown by the comparison of Treg in cocultures with WT RAW264.7 or CD137L^−/−^ RAW cells (Figure 1B). Since Treg express higher levels of CD137 than Tcon, one would expect that Treg can downregulate CD137L on RAW264.7 cells more efficiently than Tcon, which was indeed the case (Figure 1C). To ascertain that the observed downregulation of CD137L is due to CD137 expression, we cocultured WT RAW264.7 cells with WT Treg or CD137-deficient (CD137^−/−^) Treg, which were isolated from spleens of WT mice or CD137^−/−^ mice, respectively. As expected, the absence of CD137 impaired the ability of Treg to downregulate CD137L on WT RAW264.7 cells (Figure 1D).

**Figure 2:**
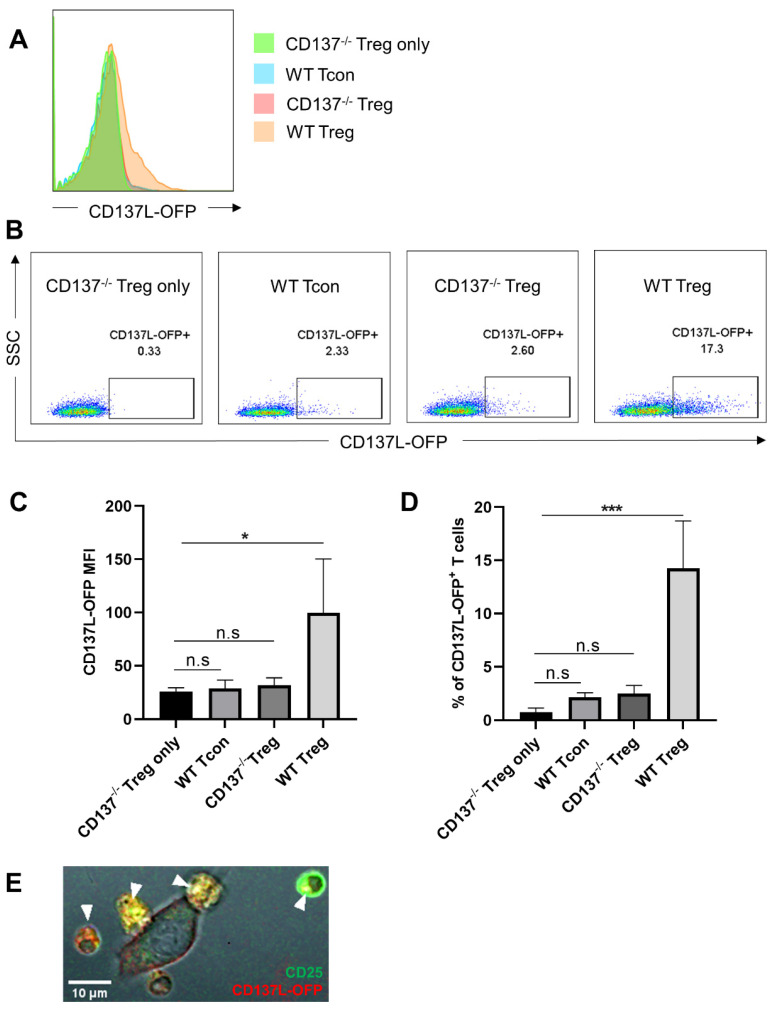
WT Treg are most effective in depleting CD137L from APC. Sorted WT Treg or WT Tcon or CD137^−/−^ Treg were cocultured with RAW-CD137L-orange fluorescent protein (OFP) cells for 1 h at a ratio of 1:1. (**A**) Cells were gated for live and single CD4^+^ cells, and CD137L-OFP levels on T cells were determined by flow cytometry. (**B**) Representative dot plots of the CD137L-OFP signal in CD137^−/−^ Treg before the coculture and in WT Treg, WT Tcon, and CD137^−/−^ Treg after the coculture. The number above the gate represents the percentage of the CD137L-OFP^+^ population. (**C**) MFI of CD137L-OFP of CD137^−/−^ Treg before the coculture and of WT Treg, WT Tcon, and CD137^−/−^ Treg after the coculture. (**D**) Percentage of CD137^−/−^ Treg before the coculture and of CD137L-OFP^+^ WT Treg, WT Tcon, and CD137^−/−^ Treg after the coculture. Data are representative of three independent experiments. Graphs show means ± SD, * *p* < 0.05, *** *p* < 0.001, n.s. not significant, one-way ANOVA with Dunnett multiple comparison test. (**E**) Confocal image of a 24 h coculture of CD25^+^ WT Treg and RAW-CD137L-OFP cells. Yellow spots indicate the internalization of CD137L-OFP (red) into CD25^+^ Tregs (green). White arrows indicate the location of the transferred CD137L-OFP in Treg.

In order to visualize the transfer of CD137L between Treg and RAW264.7 cells, we transfected an OFP-tagged CD137L into CD137L^−/−^ RAW264.7 cells (Supplementary Figure S1B). These RAW-CD137L-OFP cells with fluorescent CD137L facilitated observations by flow cytometry and confocal imaging. Coculture of these RAW-CD137L-OFP cells with WT Treg, WT Tcon, or CD137^−/−^ Treg demonstrated that WT Tregs were the most effective in depleting CD137L from RAW-CD137L-OFP cells, due to their higher level of CD137 expression. This depletion of CD137L was specific since Treg from CD137^−/−^ mice could not extract CD137L from RAW-CD137L-OFP cells (Figure 2A,B). WT Treg had the highest OFP fluorescence signal after the coculture (Figure 2C,D). The transferred CD137L-OFP was internalized by WT Treg (Figure 2E).

### 3.2. Human Treg Use CD137-Mediated Trogocytosis to Downregulate CD137L on APC

**Figure 3:**
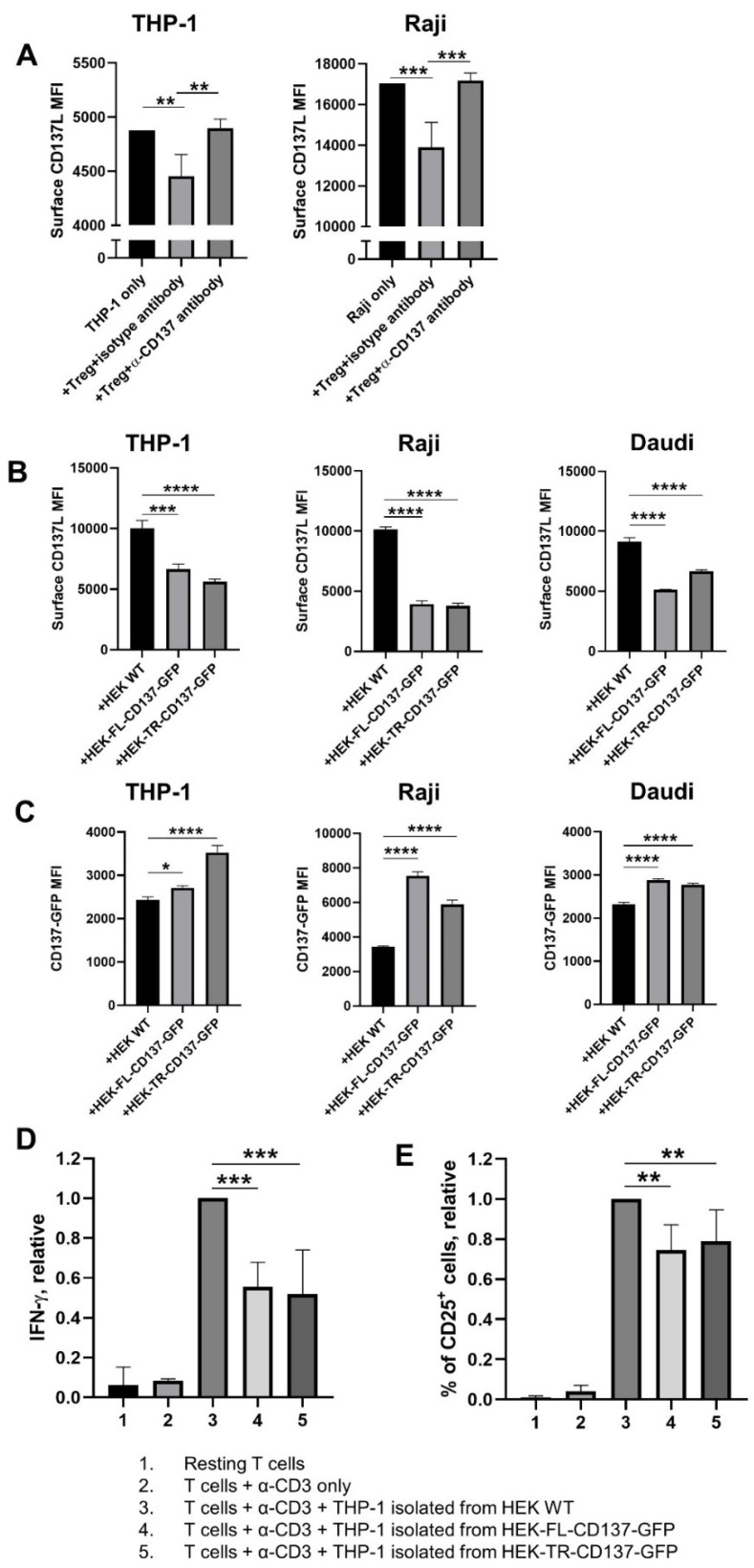
Activated human Treg and CD137-transduced HEK293 (HEK) cells use CD137 to downregulate CD137L on APC cells. (**A**) Treg isolated from human peripheral blood mononuclear cells (PBMC) from healthy donors were activated with 100 ng/mL Phorbol 12-myristate 13-acetate (PMA) and 1 µg/mL ionomycin for 24 h and washed and cocultured with Carboxyfluorescein-succinimidyl-ester (CFSE)-labelled THP-1 or Raji cells for 1 h at a ratio of 2 Treg: 1 APC, with the addition of 5 µg/mL of isotype antibody (clone MOPC-21) or anti-CD137 antibody (clone BBK-2). (**B**,**C**) THP-1, Raji, and Daudi cells after coculture with transduced HEK cells. HEK WT, HEK293 cells expressing green fluorescent protein (GFP)-tagged full-length (HEK-FL-CD137-GFP), or HEK293 cells expressing GFP-tagged truncated CD137 (HEK-TR-CD137-GFP) cells were cocultured with APC for 1 h at a ratio of 10 HEK: 1 APC. (**B**) Surface CD137L levels. (**C**) GFP fluorescence of THP-1, Raji, and Daudi cells after the coculture. (**D**,**E**) Fixed isolated THP-1 cells were cocultured with isolated CD8^+^ T cells from PBMC of healthy donors at a ratio of 1 THP-1 cell: 10 CD8^+^ T cells, for 72 h, in the presence of 0.5 µg/mL anti-CD3 antibody (clone OKT3). (**D**) IFN-γ level, relative. (**E**) Surface CD25 level, relative. Cells were analysed by flow cytometry for surface CD137L levels after gating for CFSE^+^ cells (**A**), live and single CD45^+^ cells (**B**,**C**), or live and single cells (**D**,**E**). Graphs show means ± SD, * *p* < 0.05, ** *p* < 0.01, *** *p* < 0.001, **** *p* < 0.0001, one-way ANOVA with Dunnett multiple comparison test. Data are representative for at least 3 different donors (**A**,**D**,**E**) or at least three independent experiments (**B**,**C**).

Unlike murine Treg or human intratumoural Treg, circulating human Treg from healthy donors expresses low level of CD137 during the resting state [21]. Thus, human Treg that were isolated from PBMC, were preactivated with Phorbol 12-myristate 13-acetate (PMA) and ionomycin for 24 h to upregulate CD137 expression, before being cocultured with the monocytic THP-1 cells or the B cell line Raji. Like murine Treg, human Treg also downregulated CD137L on APC (Figure 3A). Furthermore, the transfer of CD137 by Treg was blocked by a monoclonal anti-CD137 antibody, demonstrating the specificity of the transfer (Figure 3A and Supplementary Figure S2).

### 3.3. The Cytoplasmic Domain of Human CD137 Is Not Essential to Downregulate CD137L on APC

In order to assess whether CD137 signalling is essential for its suppressive effect on Treg, we generated a truncated form of human CD137 that lacks the cytoplasmic domain. We then cocultured the human APC cell lines THP-1, Raji, and Daudi with HEK293 cells expressing GFP-tagged full-length (HEK-FL-CD137-GFP) or truncated CD137 (HEK-TR-CD137-GFP). WT HEK293 cells were used as a negative control. APC in coculture with HEK-FL-CD137-GFP cells displayed a significant decrease in cell surface CD137L, compared to APC that were cocultured with WT HEK293 cells (Figure 3B). Concomitantly, the APC became positive for GFP, suggesting that they had acquired CD137-GFP and that the transfer is bidirectional (Figure 3C). The cytoplasmic domain of CD137 was not required for the transfer of either CD137 or CD137L since the same pattern was observed with cells expressing a truncated human CD137 (Figure 3B,C). Similar data, demonstrating the expendability of the cytoplasmic CD137 domain for downregulation of CD137L were obtained for murine CD137 (Supplementary Figure S3).

### 3.4. APC with Downregulated CD137L Have a Decreased Capacity to Costimulate T Cells

We hypothesized that the monocytic THP-1 cells are less effective in costimulating T cells if their CD137L level is decreased upon contact with CD137^+^ cells. To test this, we used THP-1 cells as the accessory cell in a T cell costimulation assay. After THP-1 cells were cocultured with HEK-WT, HEK-FL-CD137-GFP, or HEK-TR-CD137-GFP cells as described in Figure 3B,C, we isolated the THP-1 cells and fixed them so that the surface CD137L expression remained unchanged. Then, we cocultured the fixed THP-1 cells with primary CD8^+^ T cells, in the presence of soluble anti-CD3 antibody. Consistent with our hypothesis, THP-1 cells isolated from the coculture with HEK-WT cells were the most effective in costimulating CD8^+^ T cells. They promoted the highest level of IFN-γ secretion (Figure 3D) and CD25 expression on T cells (Figure 3E).

## 4. Discussion

This study describes a new mechanism of how Treg quench immune responses. CD137 expressed at high levels by Treg, forms a complex with CD137L on APC, after which the complex gets internalized into either Treg or APC, thereby depriving the APC of the T cell costimulatory CD137L. The transfer is bi-directional, because Treg as well as APC can internalize the CD137-CD137L complex.

Signalling by CD137 is not required for this activity, as a CD137 isoform without a cytoplasmic domain is equally capable of downregulating CD137L. However, this does not exclude CD137 signalling contributing to the inhibitory effect of Treg. Equivalent data were obtained with human and murine Treg demonstrating that this inhibitory mechanism is conserved between the two species.

Since CD137L expression on primary APC is generally low, we used APC cell lines, which have higher CD137L levels, to more clearly show the downregulation of CD137L by Treg-expressed CD137. However, we have shown in earlier studies, that CD137 trogocytosis also downregulates CD137L on primary APC [22,23].

This immunoinhibitory mechanism of CD137 on Treg parallels the function of CTLA-4 on Treg. Treg use CTLA-4 to extract the costimulatory molecules CD80 and CD86 from APC and internalize and lysosomally degrade them, thus reducing the T cell stimulatory activity of APC [20,24]. Regulation by transendocytosis or trogocytosis has also been shown for CD40L and CD30, other members of the TNF and TNF receptor families, respectively [25,26]. It would be interesting to investigate whether these molecules are also utilized by Treg to limit immune responses.

Agonistic anti-CD137 antibodies are being tested for tumour immunotherapy and their mechanism of action is believed to be costimulation of T cells. Our data show that in addition to costimulation, CD137-targeting antibodies may also enhance immune responses by other mechanisms: (1) by inhibiting the CD137-mediated downregulation of CD137L on APC and/or (2) by inducing ADCC in Treg.

We show, here, that CD137L internalization can lower the costimulatory capacity of APC, leading to reduced T cell activation. This may be one mechanism by which Treg enhance tumour growth. For other immunological conditions, such as autoimmune diseases, the impact of CD137-CD137L trogocytosis depends on the type of immune response that is the main driver of the autoimmune disease. Since the CD137-CD137L interaction mainly promotes a type 1 helper T cell response, CD137L downregulation by trogocytosis potentially ameliorates autoimmune diseases that are characterized by a Th1 polarization such as type 1 diabetes [27]. In contrast, CD137L downregulation would be expected to exacerbate autoimmune diseases with a strong Th17 component, such as lupus [28,29].

The findings of this study, the downregulation of the costimulatory CD137L through CD137 expressed by Treg, add a new mechanism to the repertoire of Treg in controlling immune responses.

## Data Availability

Not applicable.

## Supplementary Materials

The following are available online at https://www.mdpi.com/2073-4409/10/2/353/s1, Figure S1. CD137L expression on RAW264.7 cells. Figure S2. Example from one donor for the downregulation of surface CD137L on Raji cells upon coculture with activated Treg cells and how anti-CD137 antibody rescues the downregulation by blocking CD137 on Treg. Figure S3. CD137 signalling is not involved in murine CD137 trogocytosis and the transfer is bi-directional.
