# Supplementary material for: Regulatory T Cells Inhibit T Cell Activity by Downregulating CD137 Ligand via CD137 Trogocytosis
Source: Cells. 2021 Feb 9;10(2):353. doi: 10.3390/cells10020353 (PMC7914903; doi:10.3390/cells10020353)
Supplement: Supplementary file 1 [file cells-10-00353-s001.pdf]

**Suppl. Fig. 1**

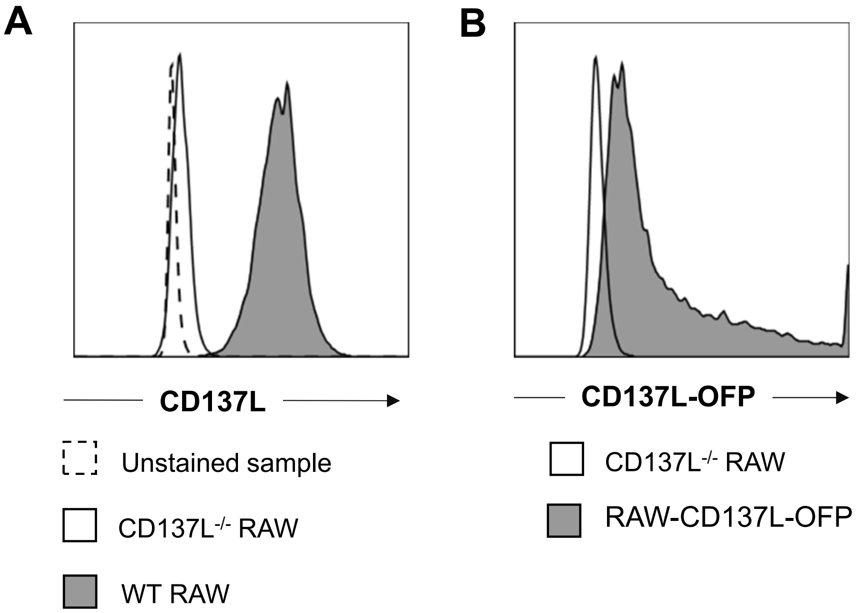


Figure S1. CD137L expression on RAW264.7 cells. (A) WT and CD137L$^{-/-}$ RAW264.7 cells were analyzed by flow cytometry for CD137L expression. (B) CD137L$^{-/-}$ RAW were transfected with CD137L-OFP to yield RAW-CD137L-OFP cells which express high level of CD137L-OFP.

**Suppl. Fig. 2**

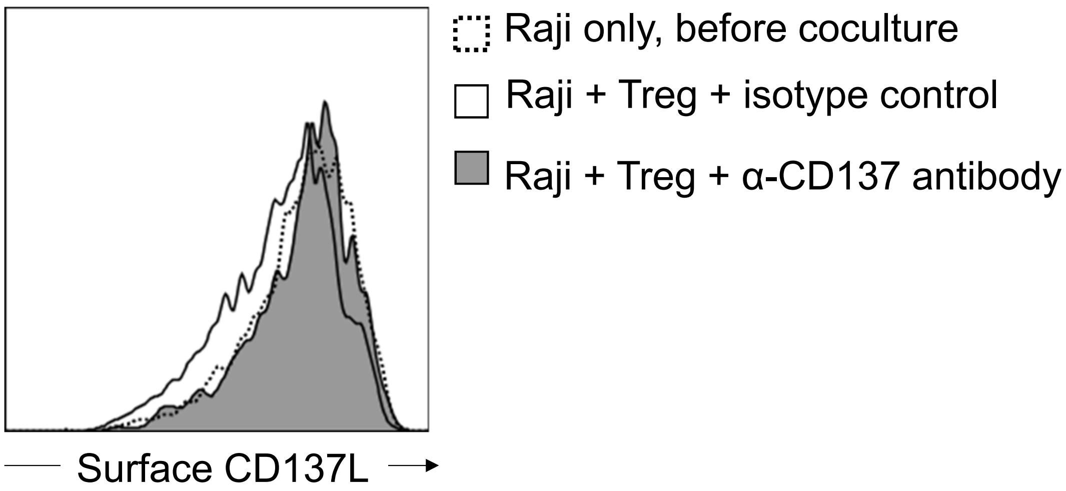


Figure S2. Example from one donor for the downregulation of surface CD137L on Raji cells upon coculture with activated Treg cells and how anti-CD137 antibody rescues the downregulation by blocking CD137 on Treg. Raji cells were labelled with CFSE before the coculture. Treg isolated from PBMC from healthy donors were activated with 100 μg/ml PMA and 1 ng/ml ionomycin for 24 h and cocultured with Raji for 1 h at a ratio of 2 Treg : 1 Raji, with the addition of 5 μg/ml of isotype antibody (clone MOPC-21), or anti-CD137 antibody (clone BBK-2). Cells were analysed by flow cytometry for surface CD137L levels after gating for single, live CFSE$^+$ cells.

## Suppl. Fig. 3

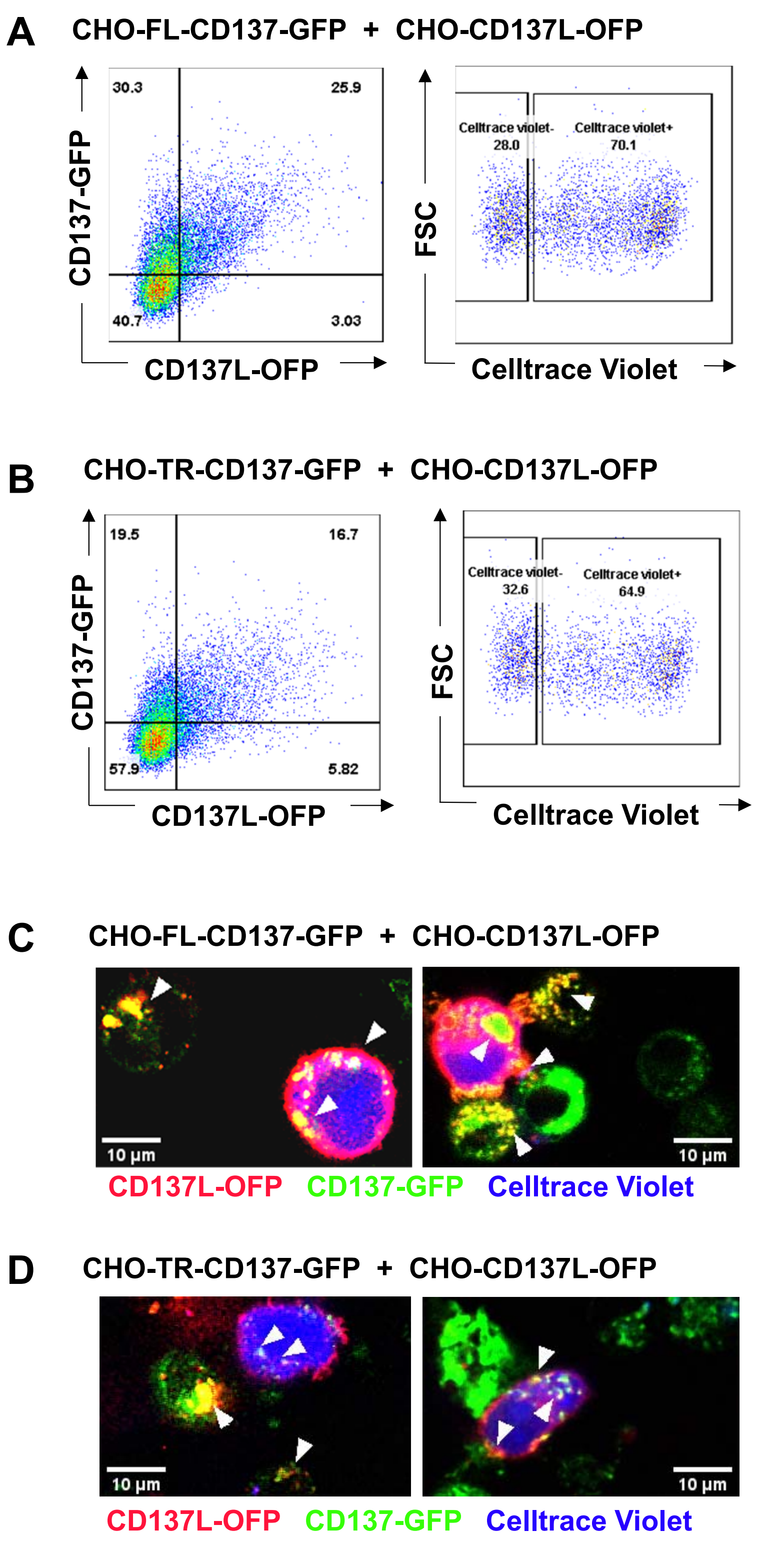


Figure S3. CD137 signalling is not involved in murine CD137 trogocytosis and the transfer is bi-directional. CHO-FL-CD137-GFP, CHO-TR-CD137-GFP or CHO-CD137L-OFP were obtained by transfecting CHO cells with the plasmid containing fluorescent murine full length CD137, truncated CD137 or CD137L, respectively. CHO-FL-CD137-GFP (A) or CHO-TR-CD137-GFP (B) cells were cocultured with CellTrace Violet-labelled CHO-CD137L-OFP cells for 3 h at a 1:1 ratio (left panel). Cells were analyzed by flow cytometry after gating for live and single cells. CellTrace Violet signals of $CD137^{+}CD137L^{+}$ double-positive cells are depicted in the right panels. Data are representative of three different experiments. (C, D). Internalisation of the CD137-CD137L complex. CHO-CD137L-OFP cells were labelled with CellTrace Violet and cocultured for 24 h at a 1:1 ratio with (C) CHO-FL-CD137-GFP cells or (D) CHO-TR-CD137-GFP cells. Yellow spots indicate the complex of CD137 (green) and CD137L (red). White arrows indicate the location of the complex in cells.
